# Emphysematous Gastritis: A Lethal Complication in a Patient With Pancreatitis

**DOI:** 10.7759/cureus.32882

**Published:** 2022-12-23

**Authors:** Bhagyasri Nunna, Pratap Parihar, Rajasbala Dhande, Gaurav Mishra, Harshith Gowda

**Affiliations:** 1 Radiodiagnosis, Jawaharlal Nehru Medical College, Datta Meghe Institute of Medical Sciences, Wardha, IND

**Keywords:** gas producing micro-organisms, intestinal perforation, computed tomography, pancreatitis, emphysematous gastritis

## Abstract

A rare illness known as emphysematous gastritis is caused by bacteria that produce gas, such as *Clostridium perfringens* and *Escherichia coli*. In gastric emphysema and emphysematous gastritis, gas can be observed within the stomach wall. Gastric emphysema should be distinguished from emphysematous gastritis. Radiological imaging features and clinical presentation are used to identify emphysematous gastritis. The imaging technique of choice for this condition is computed tomography (CT). Emphysematous gastritis has a high risk of morbidity and mortality; hence, early diagnosis and care are crucial. We discuss a case of pancreatitis with emphysematous gastritis in a male presenting to the general surgery department with abdominal pain and vomiting. The patient was advised to undergo an ultrasound and computed tomography for further evaluation.

## Introduction

As a sequela of pancreatitis, we present a case of emphysematous gastritis [1], which is a rare form of gastritis caused by micro-organisms that produce gas like *Escherichia* *coli*, *Pseudomonas aeruginosa*, *Clostridium perfringens*, *Clostridium welchii*, *Staphylococcus aureus*, and Enterobacter species [2,3]. Abnormal alcohol use, diabetes mellitus, renal failure, recent abdominal surgery, consumption of caustic substances, and usage of non-steroidal anti-inflammatory medicines are all risk factors for emphysematous gastritis [4]. It has a mortality rate of 55-60% [4,5]. A computed tomography (CT) scan is used to diagnose this condition, where we can see intramural gas in the stomach [6,7]. Early diagnosis of emphysematous gastritis followed by surgical intervention will save the patient from going into multiorgan dysfunction. In the present case, the patient went into multiorgan dysfunction, which can be avoided by early diagnosis and surgical intervention.

## Case presentation

Patient information

A 42-year-old male presented to the department of general surgery with complaints of abdominal pain and vomiting for 12 days. The patient has been an alcoholic for 10 years. There was no history of trauma.

Clinical findings

On examination, the pain was more in the epigastric region and increased on bending forward. Also, the patient is vomiting.

Diagnostic assessment

**Figure 1:**
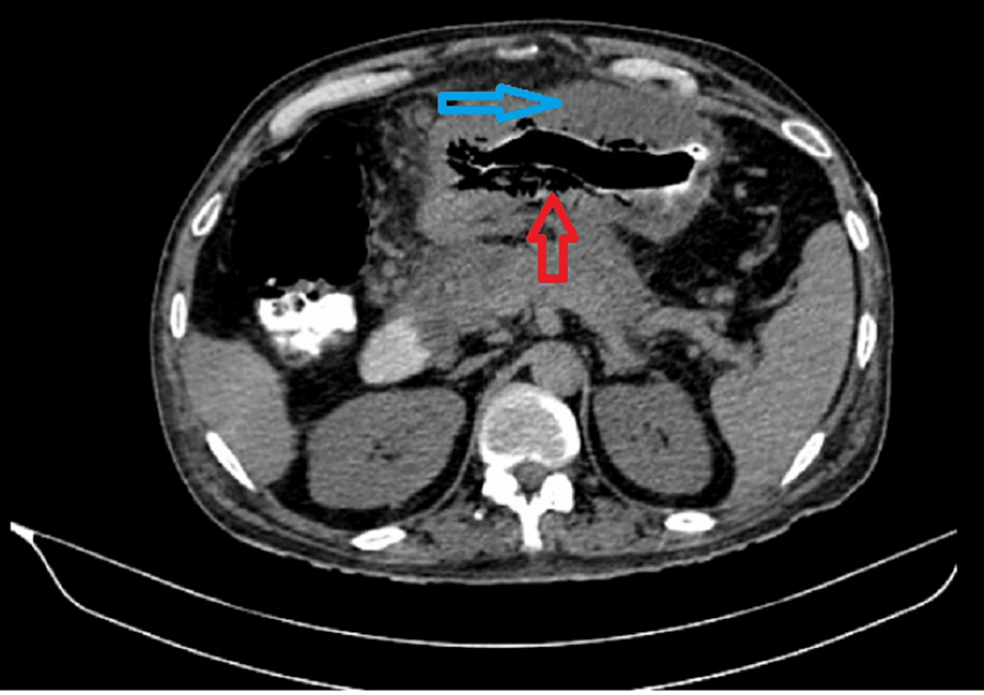
Shows diffuse wall thickening of the body of the stomach and pockets of air in the stomach wall.

**Figure 2:**
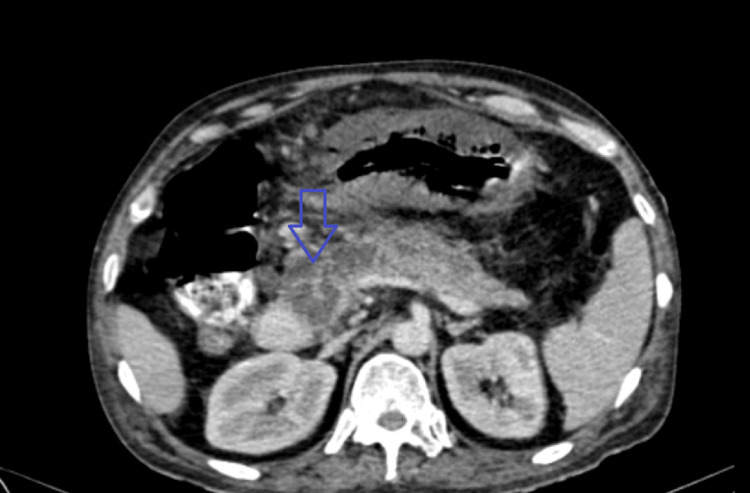
Shows multi-cystic lesion in the head of the pancreas.

**Figure 3:**
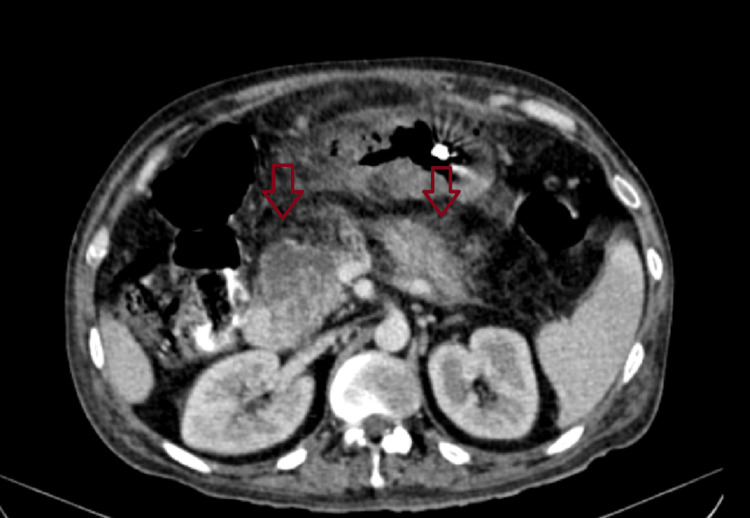
Shows minimal fat stranding around the pancreas.

As the patient is complaining of abdominal pain, an ultrasound was done in which acute pancreatitis and cholelithiasis were diagnosed. Lab investigations revealed elevated serum amylase (180 U/L) and lipase (320 U/L) levels, favoring pancreatitis. The patient was managed conservatively, and they have done a contrast-enhanced computed tomography (CECT) abdomen was to confirm pancreatitis, which showed diffuse wall thickening of the body of the stomach and pockets of air in the wall of the stomach, which is shown in Figure 1, suggestive of emphysematous gastritis; multi-cystic lesion in the head of the pancreas shown in Figure 2, suggestive of pancreatic pseudocyst; and minimal fat stranding around the pancreas shown in Figure 3, suggestive of pancreatitis. Conservative management was done, but the patient had massive hematemesis, so an upper GI endoscopy was done, which revealed diffuse stomach ulcers and an antrum exhibiting possible sealed perforation. A repeat CT revealed acute pancreatitis, emphysematous gastritis, adhesions between the stomach and pancreas, small pockets of air in the peritoneal cavity, fatty liver, and cholelithiasis. After that, the patient complained of melena, so a colonoscopy was performed, which revealed that the transverse colon was visible, the mucosa appeared normal, and a significant defect was noted communicating with a necrotic cavity.

Therapeutic intervention

According to colonoscopy findings, an emergency exploratory laparotomy was done in which the patient's distal 2/3 of the stomach was discovered to be necrosed, the pancreas' uncinate process was completely necrosed, there was pus in the paraduodenal area, the pancreas and stomach were necrosed, gastrojejunostomy and jejunojejunostomy (Billroth 2) were performed, and on the third postoperative day, he went into multiple organ dysfunction.

## Discussion

Emphysematous gastritis is a rare complication following pancreatitis, previous abdominal surgery, alcohol abuse, and renal failure. The population at risk for emphysematous gastritis includes diabetics, chronic obstructive pulmonary disease (COPD), renal failure, immunocompromised individuals, recent abdominal surgery patients, pancreatitis, and non-steroidal anti-inflammatory drug (NSAID) usage. There are two factors responsible for this condition: one is continuing sepsis within the pancreatic bed, which can predispose to thrombosis in the celiac axis, and the other is the use of inotropes [2]. Late complications of emphysematous gastritis are stricture and perforation [3], as in our case, where surgery is an absolute indication. It comes under the subtype of phlegmonous gastritis [4] and is caused by organisms that produce gas, such as *Pseudomonas*, *E. coli*, and *Clostridium welchii*. The gastric mucosa has a strong blood supply and a low pH, all of which provide an infection-resistant barrier that is destroyed when an infection occurs. Emphysematous gastritis symptoms include abdominal pain, leucocytosis, and visualizing air in the stomach wall and portal venous system on CT imaging. There is no optimal treatment for this emphysematous gastritis, though many studies suggest conservative management, not in every case it is sufficient, in case of complications like perforation one and the only management is surgical management. The patient is managed conservatively by giving broad-spectrum antibiotics, bowel rest, and nutritional management [5]. In this case, the patient developed complications like perforation, so surgical management is compulsory. The differential diagnosis for this condition should be distinguished from gastric emphysema, which is brought on by mechanical forces, including nasogastric (NG) tube insertion, emesis, alveolar leaks in COPD, chemotherapy-induced mucosal ischemia, pseudomonas infection, and appearance of a round gas bubble on radiographic imaging [6]. This condition also resembles cystic pneumatosis/cystic emphysema, in which the stomach wall will have many 1-2 mm gas-filled cysts. They typically begin with minor digestive problems [6], whereas intramural gas is seen in the stomach in cases of emphysematous gastritis [7].

## Conclusions

Emphysematous gastritis is a lethal complication that will occur following many predisposing factors; in our case, it is following pancreatitis and ended in the loss of a patient, so first and foremost, we should differentiate it from gastric emphysema, and the next step is we should check for any complications like perforation; if there are any complications, the patient should be operated on immediately, but in some conditions like in our case even though surgical intervention is done, we couldn't save the patient, so emphysematous gastritis is a lethal condition with bad prognosis. If the patient has no complications, we can manage the patient by conservative management, but its role is limited. So, the management depends on the symptoms of the patient, there is no standard treatment as such.
